# Risk of miscarriage in women with endometriosis undergoing IVF fresh cycles: a retrospective cohort study

**DOI:** 10.1186/s12958-019-0463-1

**Published:** 2019-02-12

**Authors:** Puyu Yang, Yang Wang, Zhangxin Wu, Ningning Pan, Liying Yan, Caihong Ma

**Affiliations:** 1 Center for Reproductive Medicine, Department of Obstetrics and Gynecology, Peking University Third Hospital, Haidian district, North garden road, No 49, Beijing, People’s Republic of China; 2 National Clinical Research Center for Obstetrics and Gynecology, Beijing, 100191 China; 3 Key Laboratory of Assisted Reproduction (Peking University), Ministry of Education, Beijing, 100191 China

**Keywords:** Endometriosis, Miscarriage, IVF, Singleton pregnancies

## Abstract

**Background:**

Endometriosis is thought to affect the effectiveness of ART by an increased risk of miscarriage. We aimed to investigate the impact of endometriosis in women achieving singleton pregnancies through IVF fresh cycles and risk of miscarriage.

**Methods:**

This retrospective cohort study included all women undergoing a first IVF cycle and achieving singleton pregnancies after fresh embryo transfer in a tertiary university hospital reproductive medical center between January 2008 and June 2016. Women with endometriosis were compared with women with no endometriosis. Women in the endometriosis group were all with a history of laparoscopy or laparotomy for endometriosis and/or with ovarian endometrioma. The control group was matched 1:2 according to age and study period.

**Results:**

Among the cohort, we identified 1006 women with endometriosis as study group and 2012 unaffected women matched in a 1:2 ratios as control group. The miscarriage rate between women with and without endometriosis was similar (22.4 and 20.1%, *P* = 0.085). The odds ratio after adjusting for the risk factors for miscarriage was 1.14 (95% confidence interval 0.95–1.37). In the study group, the women with and without endometrioma did not show a significant risk of miscarriage, (19.8 and 23.8%, *P* = 0.152, OR 0.79, 95% CI 0.58–1.09). The miscarriage rate in women with endometrioma ≥30 mm (37.3 ± 7.1 mm) and < 30 mm (19.3 ± 5.5 mm) was not significantly different, (24.7 and 18.5%, *P* = 0.229, OR 1.44, 95% CI 0.79–2.63). After adjustment for risk factors for miscarriage, the presence of endometrioma and the size of endometrioma, regression model confirmed no significant increase for the risk of miscarriage in the subgroup analyses.

**Conclusions:**

The risk of miscarriage did not statistically increase in women with endometriosis who achieved pregnancy through IVF fresh cycles.

## Introduction

Endometriosis is one of the most important benign gynecologic diseases in reproductive aged women [1] and approximately 25–40% of women with endometriosis diagnosed with infertility [2]. It has a negative impact on fertility through various mechanisms that interfere with the entire reproductive process, such as sperm-oocyte interaction, ovarian reserve, implantation, depending on the tissue in which it is exerted [3].

During the last few years, the developments and progress in assisted reproductive technology (ART) treatments have led to an increasing number of successful pregnancies in women with endometriosis [4]. The pre-pregnancy uterine milieu plays as a risk factor of placental bed disorders [5]. Eutopic endometrium in patients with endometriosis is abnormal and obstetric research has shown that abnormal endometrium [6], its decidualized stroma and impaired placentation, leads to preterm birth, intrauterine growth restriction, and babies’ small for gestational age [7]. There are several reasons why endometriosis may contribute to adverse pregnancy outcomes [8], including endometrial resistance to selective actions of progesterone, inflammatory processes at the endometrial and systemic levels, inadequate uterine contractility, and endometrial excessive activation of free radical metabolism [9]. Research on the pathogenesis of poor pregnancy outcomes suggests that all these alterations of the local endometrial environment may associated with a risk of preterm labor, fetal growth restriction, and placental disorders [10].

Miscarriage is the most common pregnancy complication in the first trimester [11]. Previous studies are controversial about the association between the miscarriage rates and endometriosis-related infertility [9]. Santulli et al. [12] found that endometriosis-affected women displayed a significantly higher rate of previous spontaneous miscarriages than endometriosis-free controls. According to the impact of endometriosis on miscarriage rate, the previous miscarriage rate was significantly higher in women with endometriosis compared with the controls (29% versus 19%, *P* < 0.001, adjusted IRR 1.70, 95% CI 1.34–2.16). However, Leonardi et al. [13] found that the number of miscarriages in women with and without endometriosis was similar (15 and 19%, OR 0.76, 95% CI 0.50–1.16) and the risk of miscarriage did not increase in women with endometriosis achieving pregnancy with the use of IVF.

Ovarian endometrioma is a common disease and may be occurred in nearly 30–40% of women with endometriosis [14]. An expanding endometrioma in pregnancy constituted a risk factor for subsequent abscess formation or rupture after oocyte retrieval. While few studies have focused on whether the size of ovarian endometrioma increased the risk of miscarriage rate. The excision of ovarian endometriomas is still a controversial and unsolved issue in this field. According to the guidelines of the European Society of Human Reproduction and Embryology (ESHRE), patients with endometriomas larger than 3 cm should not undergo laparoscopic cystectomy before IVF to improve pregnancy rate [15]. However, several studies showed that removal of endometriomas in women with endometriosis often improved reproductive outcomes [16].

Though compelling clinical evidence of an association between endometriosis, the size of endometrioma and subsequent miscarriage is still lacking, we hypothesized that miscarriage should be increased in patients with ovarian endometrioma. We tested this hypothesis by examining whether miscarriage was more likely in women with endometriosis who required IVF than in other IVF patients. In the subgroup analysis, we compared the miscarriage rate between women with and without endometrioma in IVF fresh cycles and investigated the miscarriage rate in different size of endometrioma subgroup.

## Materials and methods

### Inclusion and exclusion of subjects

We performed a retrospective cohort study from our electronic database between January 2008 and June 2016 at the Reproductive Medicine Center of Peking University Third Hospital. All women with undergoing first IVF cycle and achieving singleton pregnancies after fresh embryo transfer were included. Exclusion criteria included: [1] aged older than 42 years; [2] hydrosalpinx, abnormal uterine cavity, excessive uterine volume and uterine malformations (presence of submucosal fibroids or uterine septum); [3] abnormal karyotype of woman or her husband; and [4] diseases with possible impact on pregnancy such as diabetes, hypertension, thyroid dysfunction and antiphospholipid syndrome. The study was approved by the Institutional Review Board at Reproductive Medicine Center of Peking University Third Hospital. All women were informed that their clinical data could be used for researches purposes. The cohort of women was separated into two groups named “endometriosis group” as study group and “non-endometriosis control group” as control group. Endometriosis was diagnosed by the laparoscopy or laparotomy. The diagnosis of ovarian endometrioma in our study was based on the finding of ovarian endometrioma on at least two pelvic ultrasound scans before IVF, typically separated by 3 months, to ensure persistence of the lesion. More specifically, ovarian endometrioma was defined as a round-shaped cystic mass with a minimum diameter of 10 mm. It was characterized by thick walls, regular margins, homogeneous low echogenic fluid content with scattered internal echoes, and without papillary projections [17]. Controls were matched to cases by age, type of cycles and study period in a 1:2 ratios. In our study, patients in the control group were categorized as tubal factor infertility if their enrollment HSG showed unilateral or bilateral fallopian tube blockage [18]. The male factor infertility included Azoospermia, severe oligosperma, asthenospermia and teratospermia.

### IVF treatment

During the IVF cycle, the patients were managed by a standardized clinical protocol as reported previously [19]. The dose of gonadotrophin was determined individually according to the woman’s age, day 3 serum follicle-stimulating hormone (FSH) value and ultrasound characteristics of the ovaries. For ovarian stimulation, the long agonist, antagonist, and short agonist protocols were performed. Thirty-six hours before oocyte retrieval, human chorionic gonadotropin (hCG) was administered when the diameter of more than 2 follicles reached to 18 mm. Day-3 or day-5 embryos were transferred in a fresh cycle with luteal support by vaginal and/or intramuscular progesterone. Serum hCG was measured 14 days after ET and was considered positive for hCG level ≥ 10 IU. Transvaginal ultrasonography at 30 days after transfer confirmed clinical pregnancy if intrauterine gestational sac was demonstrated. Miscarriage rate was defined as loss of clinical pregnancy before 24 weeks of gestation divided by the number of clinical pregnancies.

### Statistical analysis

All data were stored in a computerized database. The data analysis was performed by using SPSS 24.0 software (IBM Corporation, Armonk, NY, USA). It was appropriate to use Chi-square test or Student’s t-test. Results were reported as odds ratio (OR) and 95% confidence interval (CI). *P* value < 0.05 was considered statistically significant. We used the 1 tail *P* value to calculate the miscarriage rate between women with and without endometriosis and use the 2-tail test to calculate the *P* value in subgroup analysis. A logistic regression model and univariate analysis was used to calculate the adjusted ORs.

## Results

**Fig. 1:**
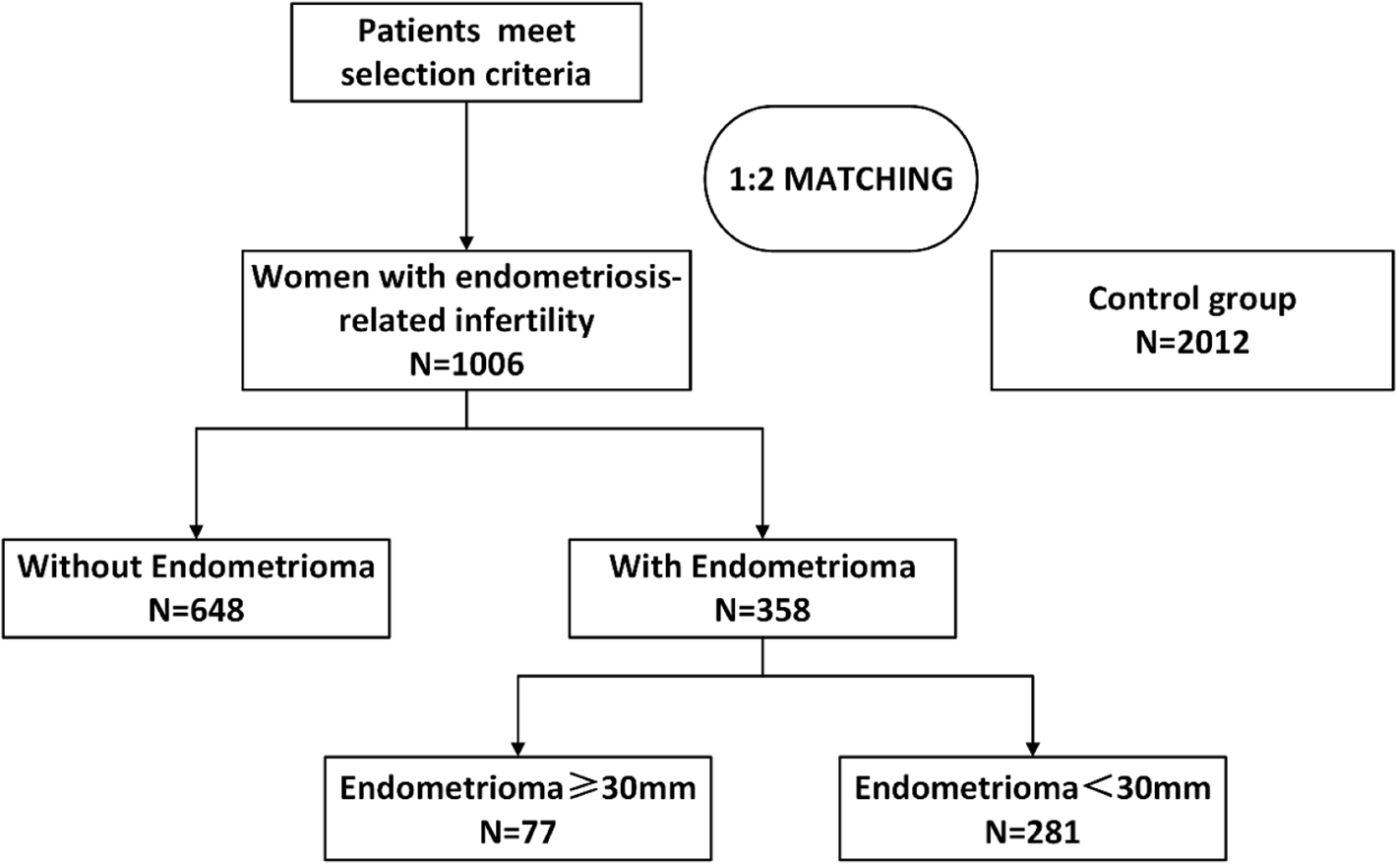
Patient inclusion flow chart

Between January 2008 and June 2016, there are 37,062 IVF stimulating cycles and 23,644 women in fresh embryo transfer cycles achieving clinical pregnancy. Fifteen thousand, eight hundred and-fourteen women were included in the analyses after getting singleton pregnancy. There were 2497 women-initiated cycles of patients with endometriosis. Among them, we identified 1006 women with endometriosis as study group and 2012 unaffected women matched in a 1:2 ratios as control group who met the inclusion and exclusion standard (Fig. 1). In the study group, 648 (64.41%) women had laparoscopy surgery to remove endometrioma and the endometriomas didn’t recur after surgery. Ninety women (8.95%) had bilateral endometriomas and 268 (26.64%) women had unilateral endometriomas, respectively. The mean ± SD diameter of the cysts was 23.1 ± 9.5 mm.

The baseline characteristics of patients in two groups are shown in Table 1. There was a statistically significant difference in the body mass index (BMI), gravidity and parity (*P* < 0.001) between the two groups. During ovarian stimulation, the study group required long time and a higher dose of gonadotropins, but retrieved fewer oocytes. Tubal factor and male factor infertility were the two most common diagnoses in the control group.

**Table 1:** Baseline characteristics of the two groups

Characteristics	Endometriosis *n* = 1006	Controls *n* = 2012	OR (95% CI)	*P* value
Female’s Age (years)	33.04 ± 3.66	32.83 ± 3.76	–	0.16
Male’s Age (years)	34.75 ± 4.72	34.40 ± 4.85	–	0.06
BMI (kg/m2)	21.94 ± 3.12	22.44 ± 3.20	–	< 0.001
Gravidity [n (%)]				
None	669 (66.5)	968 (48.1)	2.141 (1.829–2.506)	< 0.001
1	206 (20.5)	501 (24.9)		
2	85 (8.4)	319 (15.9)		
≥ 3	46 (4.6)	224 (11.1)		
Duration of infertility (years)	4.77 ± 3.38	4.98 ± 3.54	–	0.13
Parity [n (%)]				
None	965 (95.9)	1805 (89.7)	2.699 (1.913–3.808)	< 0.001
1	41 (4.1)	187 (9.3)		
2	0 (0)	20 (1.0)		
Day 3 serum FSH (IU/ml)	7.10 ± 3.64	6.98 ± 2.75	–	0.39
Indication to IVF				
Endometriosis	719 (71.47)			
Endometriosis +Tubal factor	287 (28.53)			
Male factor		256 (12.72)		
Tubal factor		1583 (78.68)		
Mixed (without endometriosis)		173 (8.60)		
Regimen of ovarian hyper-stimulation				
Long protocol	719 (71.5)	1244 (61.8)	1.547 (1.313–1.822)	< 0.001
Flare-up protocol	95 (9.4)	434 (21.6)		
GnRH antagonists	186 (18.5)	328 (16.3)		
Other	6 (0.6)	6 (0.3)		
Duration of stimulation (days)	11.72 ± 2.53	11.36 ± 2.44	–	< 0.001
Total dose of FSH administered (IU)	3467.85 ± 1448.45	2850.55 ± 1267.95	–	< 0.001
Estradiol at the time of hCG administration (pg/ml)	2247.77 ± 1601.71	2823.35 ± 1882.31	–	< 0.001
Total oocytes retrieved	10.07 ± 5.64	12.36 ± 6.02	–	< 0.001
Stage at embryo transfer				
72 h	977 (97.1)	1972 (98.0)	0.683 (0.421–1.109)	0.12
Blastocysts	29 (2.9)	40 (2.0)		
Miscarriage	225 (22.4)	405 (20.1)	1.143 (0.951–1.374)	0.09

Data are expressed as Mean + 1 SD or number (percentage) as appropriate

Data are compared using unpaired Student’s t test or Fisher’s exact test

Characteristics of women with and without endometrioma in fresh cycles are shown in Table 2. The women with endometrioma were preferred to use long protocol, required long time and a higher dose of gonadotropins, but retrieved fewer oocytes. The levels of estradiol at the time of hCG administration were similar in the two groups. The total dose of FSH administered and the stimulation time length were significantly higher in women with endometrioma.

**Table 2:** Characteristics of the fresh cycles in the two study groups

Characteristics	Endometriosis with Endometrioma *n* = 358	Endometriosis without Endometrioma *n* = 648	OR (95% CI)	*P* value
Duration of stimulation (days)	12.14 ± 2.37	11.49 ± 2.59	–	< 0.001
Total dose of FSH administered (IU)	3706.35 ± 1472.86	3336.09 ± 1418.81	–	< 0.001
Estradiol at the time of hCG administration (pg/ml)	2240.06 ± 1459.17	2252.25 ± 1680.12	–	0.91
Total oocytes retrieved	9.78 ± 5.21	10.23 ± 5.87	–	0.22
In vitro fertilization technique used				
IVF	254 (70.9)	466 (71.9)	0.954 (0.717–1.269)	0.75
ICSI	104 (29.1)	182 (28.1)	–	
Number of embryos-blastocysts transferred				
1	37 (10.3)	79 (12.2)	0.830 (0.549–1.256)	0.38
≥ 2	321 (89.7)	569 (87.8)		
Stage at embryo transfer				
72 h	344 (96.1)	633 (97.7)	0.582 (0.278–1.220)	0.15
Blastocysts	14 (3.9)	15 (2.3)		
Miscarriage	71 (19.8)	154 (23.8)	0.794 (0.578–1.089)	0.15

Table 3 shows miscarriage rate in relation to the size of ovarian endometrioma, respectively. The miscarriage rate in women with endometrioma < 30 mm (19.3 ± 5.5 mm) and ≥ 30 mm (37.3 ± 7.1 mm) was not significantly different, (18.5 and 24.7%, respectively, *P* = 0.229). The group of patients with larger endometriomas (≥30 mm) was comparable with the group of women with smaller endometriomas (< 30 mm) in terms of demographic data. During ovarian stimulation, the women with larger endometriomas (≥30 mm) were required long time and a higher dose of gonadotropins, but retrieved fewer oocytes. According to the size of endometriomas by quartile, we divided the groups as Q1(< 16.0 mm), Q2 (≥16.0 mm and < 21.5 mm), Q3(≥21.5 mm and < 28.1 mm) and Q4(≥28.1 mm). The miscarriage rate was 12.2, 20.4, 21.3 and 24.7%, there was no significant difference between the four groups (*P* = 0.213).

**Table 3:** Baseline characteristics of the fresh cycles according to endometrioma size

Characteristics	Endometrioma≥30 mm(*n* = 77)	Endometrioma< 30 mm (*n* = 281)	OR (95% CI)	*P* value
Female’s Age (years)	32.22 ± 3.50	32.77 ± 3.36	–	0.21
BMI (kg/m2)	21.18 ± 2.53	22.18 ± 3.39	–	0.02
Duration of infertility (years)	4.48 ± 3.64	4.31 ± 3.13	–	0.69
Duration of stimulation (days)	12.62 ± 2.75	12.01 ± 2.24	–	0.04
Total dose of FSH administered (IU)	4042.37 ± 1599.39	3614.27 ± 1425.47	–	0.02
Estradiol at the time of hCG administration (pg/ml)	2180.29 ± 1475.85	2256.07 ± 1456.92	–	0.69
Total oocytes retrieved	9.32 ± 5.21	9.91 ± 5.21	–	0.39
Miscarriage	19 (24.7)	52 (18.5)	1.443 (0.792–2.627)	0.23

The miscarriage rate between women with and without endometriosis was similar, (22.4 and 20.1%, respectively, *P* = 0.085). The odds ratio adjusted for body mass index, parity, age, duration of infertility and male factor infertility was 1.14 (95% confidence interval 0.95–1.37). In the study group, the women with and without endometrioma did not show a significant risk of miscarriage, (19.8 and 23.8%, *P* = 0.152, OR 0.79, 95% CI 0.58–1.09). The miscarriage rate in women with endometrioma ≥30 mm (37.3 ± 7.1 mm) and < 30 mm (19.3 ± 5.5 mm) was not significantly different, (24.7 and18.5%, *P* = 0.229, OR 1.44, 95% CI 0.79–2.63). The results from the univariate and multivariate regression analyses were summarized in Table 4. After adjustment for risk factors for miscarriage, the presence of endometrioma and the size of endometrioma, regression model confirmed no significant increase for the risk of miscarriage in the subgroup analyses.

**Table 4:** Subgroup analyses on the risk of miscarriage

	Subgroup	Abortions n (%)	Subgroup	Abortions n (%)	*P* value	Crude OR (95% CI)	Adjusted OR (95% CI)
In vitro fertilization technique used-IVF	Endometriosis		Controls				
	720	154 (21.4)	1557	300 (19.3)	0.239	1.140 (0.917–1.418)	1.099 (0.869–1.391) ^a^
In vitro fertilization technique used-ICSI	Endometriosis		Controls				
	286	71 (24.8)	455	105 (23.1)	0.586	1.101 (0.779–1.555)	1.186 (0.815–1.727) ^a^
Endometriomas at ultrasound	Yes		No				
	358	71 (19.8)	648	154 (23.8)	0.152	0.794 (0.578–1.089)	0.883 (0.634–1.222) ^b^
Endometrioma size	≥ 30 mm		< 30 mm				
	77	19 (24.7)	281	52 (18.5)	0.229	1.443 (0.792–2.627)	1.412 (0.767–2.600) ^b^

a. Model adjusted for BMI, gravidity, parity, male factor infertility, total dose of FSH administered and number of oocytes retrieved

b. Model adjusted for BMI, duration of stimulation and total dose of FSH administered

## Discussion

This retrospective study focused on the miscarriage rate in women with endometriosis by analyzing a large database. We found that there was an increased risk of miscarriage in women with endometriosis but no significant difference between patients with and without endometriosis (*P* = 0.154). Our results showed that the risk of miscarriage did not increase in women with endometriosis achieving singleton pregnancies through IVF fresh cycle and reported that women with endometriomas did not display significantly higher rate of miscarriage in the subgroup analyses.

Previous studies on the miscarriage of endometriosis-associated infertility have shown similar results. Hamdam et al. [20] conducted a meta-analysis to compare the ART outcomes in women with and without endometriosis. The result of miscarriage did not show a significant difference in miscarriages between women with and without endometriosis (OR 1.26, 95% CI 0.92–1.70). Comadran et al. [21] performed a retrospective cohort study including women undergoing IVF and investigated that there was no significant difference between endometriosis-associated infertility group and tubal factor control group in terms of miscarriage rate (*P* = 0.623, OR 1.048, 95% CI 0.867–1.269). To some extent, one advantage of our study was that the results were based on a large sample size and we analyzed in the characteristics of endometriosis and size of endometriomas. Maggiore et al [9]showed that there was no sufficient evidence supporting an association between endometriosis and miscarriage according to the current studies in a systematic review on endometriosis patients during pregnancy. Senapati et al. [22] conducted a population-based retrospective cohort study cycles to evaluate the clinical outcome on the impact of endometriosis on IVF. They compared the women with endometriosis and tubal factor in fresh and frozen cycles, and found that there was no significant difference in the incidence of miscarriage. These results strongly supported our opinion.

In contrast, some previous reports on the outcome of miscarriages for endometriosis-associated infertility have showed different results. Kohl’s study [23] suggested that mild endometriosis was a superficial lesion related to inflammatory disorder, which possibly lead to defective fertilization and implantation and increase the risk of miscarriage. Saraswat et al. [24] performed a national population-based cohort study to determine pregnancy outcomes in women with endometriosis. When compared to women without endometriosis, women with endometriosis had a significantly higher risk of miscarriage (OR1.76, 95%CI 1.44–2.15), respectively. Hjordt et al. [25]found increased risk for miscarriages (OR 1.2, 95% CI 1.2–1.3) among endometriosis-affected women based on the data of national Danish registries. Moreover, two systematic reviews investigated the impact of endometriosis on ART outcomes. Barbosa et al. [26] reviewed 92 studies and found that the risk of miscarriage increased among pregnant women with endometriosis (OR 1.31, 95%CI 1.07–1.59). In a more recent meta-analysis, Zullo et al. [27] included 24 studies and reported that women with endometriosis had a statistically significantly higher risk of miscarriage (OR 1.75, 95% CI 1.29–2.37). In the meta-analysis, most outcomes had very high statistical heterogeneity. The findings should be interpreted with caution due to the very low quality of the available evidence. Changes in diagnosis and treatment of endometriosis, obstetric care, laparoscopic equipment and techniques, and uptake of assisted reproduction could have influenced the outcomes evaluated. Considering the limit heterogeneity caused by the advancement in surgical equipment and assisted conception technology, it is better to assess the outcomes only on studies published after 2000. Previous studies have shown strong associations between age and a higher risk of miscarriage [28]. In our study, the patients included in the two groups were all matched 1:2 according to age and the matched-age was very important and appropriate.

**Fig. 2:**
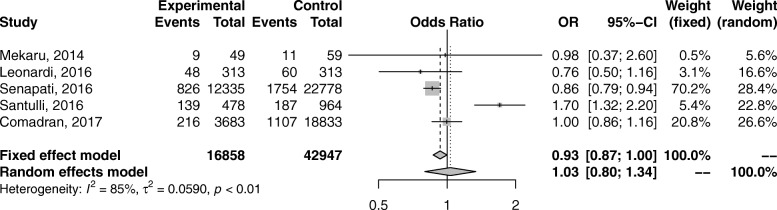
Forest plot showing individual and combined effect size estimates and 95% confidence intervals (CIs) in studies that evaluated the risk of miscarriage in women with endometriosis compared with a control group of women without endometriosis. Horizontal lines indicate 95% CIs; boxes show the study-specific weight; diamond represents combined effect size; dashed line indicates the overall estimate

**Fig. 3:**
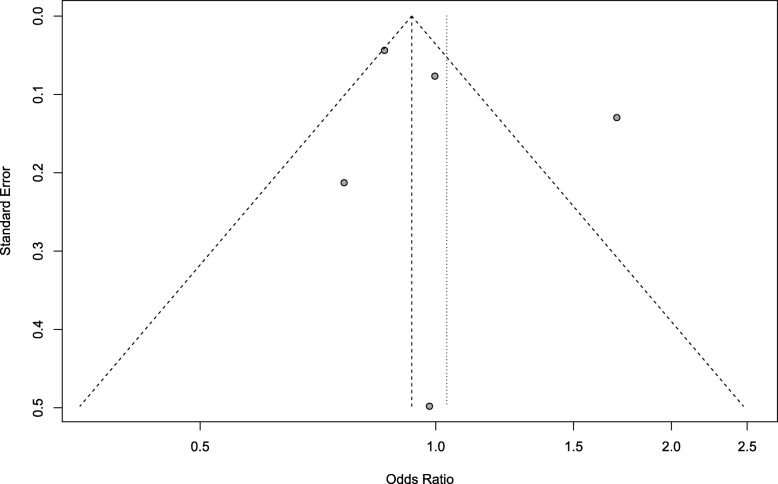
Funnel plot for the risk of miscarriage, LNP (log of proportion) represented in the x-axis and standard error of log proportion in the y-axis

Several observational nonrandomized studies evaluating the effect of endometriosis on miscarriage rate have been published, so far with conflicting results. Our systematic review and meta-analysis evaluated the effect of endometriosis on the risk of miscarriage. We included seven studies from 2014 to 2017 in recent four years reporting the risk of miscarriage rate in women with endometriosis compared with a control group of women without endometriosis. The meta-analysis was performed using the random effects model of DerSimonian and Laird, to produce summary treatment effects in terms of OR with 95% confidence interval (CI). Heterogeneity was measured using I-squared (Higgins I [2]). *P* < 0.05 was considered statistically significant. This meta-analysis, from 7 studies [12, 13, 21, 22, 24, 25, 29], including 196,795 women, evaluated the effect of endometriosis on the risk of miscarriage. Compared with the control group, no statistical differences were found in the incidence of miscarriage (OR 1.24; 95% CI, 0.93–1.64) (Figs. 2 and 3). We observed no statistically different in the risk of miscarriage in women with endometriosis. This finding should be interpreted with caution because the evidence was deemed to be of very low quality.

As far as is known, inconsistencies in reports on the impact and management of ovarian endometriomas in assisted reproduction treatment deserved discussion. Some previous studies believed that endometriomas > 30 mm should be treated surgically before ART treatments, whereas others argued that the surgery could significantly damage ovarian reserve and consequently compromised success [30]. Based on our findings, the presence of endometrioma did not affect the risk of miscarriage. In particular, we found that the miscarriage rate was very similar between the women with endometriomas (≥30 mm) and endometriomas (< 30 mm). In a recent study, the presence of ovarian endometriomas did not affect oocyte developmental competence in women undergoing IVF [31]. Benaglia et al. [32] found that women with endometriomas achieved pregnancy through IVF did not seem to have a significant increased risk of obstetrical complications. The live birth rates in women with and without endometriomas were no statistically different (78% vs 83%, *P* = 0.39, OR 0.79, 95%CI 0.38–1.68). He also reported that the presence of small endometriomas (< 40 mm) at the time of IVF did not markedly affect responsiveness to hyperstimulation [33]. In contrast, Coccia et al. [34] recently observed the endometrioma size of 30 mm might influence the response of the diseased ovary and suggested that surgery before ART treatment might be a valuable option in patients with endometriomas > 30 mm. The current studies that have evaluated the association between endometriomas and miscarriage were controversial. Our results demonstrated that there was no statistically different in miscarriage rate between the women with endometriomas compared with those without endometriomas (19.8 and 23.8%, respectively, *P* = 0.152, OR 0.79, 95% CI 0.58–1.09).

Over the past few years, the mechanism of endometriosis-related infertility and its impact on poor IVF reproductive outcome has not been fully established [35]. The process of endometriosis could affect the oocyte quality, quantity and endometrial receptivity with inflammatory factor [36]. Endometriosis is reported to be associated with different mechanisms, including endocrine balance, molecular and functional mechanism, changes of the hormonal milieu [37]. There is biological evidence showing that the endometrium of women with endometriosis differs from the endometrium of healthy unaffected women. Differences have been found in stem cell content, hormone sensitivity, cellular proliferation, adhesion, invasiveness, angiogenesis and immune modulation [38]. Briefly, in endometriosis there is an abnormal gene expression; a local estrogen production and altered endometrial response to progesterone; an increased nerve density and oxidative stress. Moreover, the metabolic and molecular abnormalities in endometriosis increase angiogenesis and proliferation, decrease apoptosis, allow local production of estrogen, create progesterone resistance and impair cytokine expression [39]. According to the present study, the process of assisted reproductive technology was different from spontaneous pregnancy. In natural conception, both the inflammatory peritoneal fluid and peritoneal inflammation milieu might affect oocyte development in some aspect [40]. On the other hand, IVF had the advantage to protect the oocyte from the peritoneal fluid. The oocyte could be far away from the harmful milieu that may cause disease in some cases. These were the possibilities that could explain our results.

However, this study had several limitations that need to be taken into consideration. First, it was not possible to find the exact surgical classification endometriosis in the analysis, since the women had surgery in different hospitals and these data were not available in the registry. Furthermore, to some extent, the sonographic diagnosis of ovarian endometrioma was not very reliable. The identified of endometriosis with ultrasound requires an active approach and utmost expertise. Sometimes we had bias and couldn’t evaluate the impact of deep endometriosis. Third, the size of ovarian endometriomas in our study was relatively small (the mean diameter was 23.1 ± 9.5 mm). Nevertheless, the mean dimension of the endometriomas in operated women was generally 40–50 mm. Therefore, we cannot rule out that larger cysts may have an impact on miscarriage rate. Further evidence was required to address this point. The sample size is also one limitation to the findings of our study. The moderate number of included patients restricts the explanatory power of this study and a pre-planned power calculation was not performed. However, it would have been difficult to recruit about five thousand women with endometriosis undergoing IVF fresh cycles in one reproductive center. Comparing with other studies, we have a larger number of patients in single medical center to investigate the impact of endometriosis in women through IVF fresh cycles and risk of miscarriage. In addition, retrospective study had limitations of study design and documentation accuracy, so it was hard to prove the hypothesis definitely. Therefore, further studies, especially prospective controlled studies with large sample sizes are necessary to confirm our findings.

## Conclusions

In conclusion, our study found that in spite of its limitations, the risk of miscarriage did not increase in women with endometriosis achieving pregnancy through IVF. Moreover, women with endometriomas do not seem to have a significant increased risk of miscarriage. However, considering the inconsistence of previous studies on this topic, evidence from larger studies and prospective analyses is required to investigate the effect of ovarian endometriomas on miscarriage rate. It is also necessary to perform basic research to better define the relationship between miscarriage and endometriosis in the future.

## Abbreviations

ART: Assisted Reproductive Technology
BMI: Body mass index
CI: Confidence interval
ESHRE: European Society of Human Reproduction and Embryology
FSH: Follicle stimulating hormone
hCG: human chorionic gonadotropin
HRT: Hormone replacement therapy
ICSI: Intracytoplasmic sperm injection
IVF-ET: In vitro fertilization and embryo transfer
OR: Odds ratio
